# Alternative treatment of polycystic ovary syndrome: pre-clinical and clinical basis for using plant-based drugs

**DOI:** 10.3389/fendo.2023.1294406

**Published:** 2024-01-11

**Authors:** Sidra Malik, Saira Saeed, Ammara Saleem, Muhammad Imran Khan, Aslam Khan, Muhammad Furqan Akhtar

**Affiliations:** ^1^ Riphah Institute of Pharmaceutical Sciences, Riphah International University, Lahore, Pakistan; ^2^ Department of Pharmacology, Faculty of Pharmaceutical Sciences, Government College University Faisalabad, Faisalabad, Pakistan

**Keywords:** phytochemical, herbal therapy, PCOS, infertility, oxidative stress, inflammation

## Abstract

The most common cause of infertility and metabolic problems among women of reproductive age is polycystic ovary syndrome (PCOS), a multifaceted disorder. It is an endocrine disorder that occurs in approximately one in seven women. Among these PCOS patients, two thirds will not ovulate on a regular basis and seek treatment for ovulation induction. The symptoms vary in their severity, namely ovulation disorders, excessive androgen levels, or polycystic ovarian morphology. All these symptoms require a therapeutic approach. Many drugs are used to eradicate PCOS symptoms, like metformin, clomiphene citrate, spironolactone, and pioglitazone. Long-term treatment is required to achieve the desired outcome, which is often accompanied by significant adverse reactions. Some herbs and phytochemicals are equally effective for treating PCOS and produce minimal side effects. Recently, herbal products are gaining popularity due to their wide biological activities, safety, availability, and efficacy. The present review covers aetiology, current treatment, pathophysiology, and detailed pre-clinical and clinical studies on plants and phytochemicals that are proven to be useful for the treatment of symptoms associated with PCOS.

## Introduction

The Stein–Leventhal syndrome was the initial name for the condition that is now known as polycystic ovary syndrome (PCOS) (1). It is a harmful illness that predominantly affects females and is characterized by an enlargement of the ovaries that are filled with many tiny cysts that are actually immature follicles (2). Ovulation, menstrual abnormalities, infertility, and insulin resistance are all related to PCOS. Acne, hirsutism, and weight gain are all possible symptoms of PCOS (3). As the condition worsens, it culminates in other health problems such as dysfunctional uterine haemorrhage, obesity, Type 2 diabetes, endometrial cancer, high cholesterol, and cardiovascular diseases (4). Modifications to a woman’s lifestyle, as well as pharmacological therapies, are currently used as standard care treatments for PCOS (5). Alterations to one’s nutrition and an increase in physical activity are the desired lifestyle changes for the treatment of PCOS (6). Even though, there are many drugs on the market, the most effective strategy for PCOS is to adjusting one’s lifestyle. Losing weight and exercising are two of the most effective ways to treat PCOS without the risk of negative side effects (7).

The use of oestrogen-progestin combinations, antiandrogens, and hypoglycaemic medications are some examples of pharmacological therapies for PCOS (8). However, their chronic use is associated with the risk of endometrial cancer in women after menopause. This risk seems to increase as the dose and the length of use increase (2). Since ancient times, medicinal plants have had a great deal of potential for treatment and mitigation of acute and chronic diseases, and now, as a result of the research that has been conducted, new medicinal plants that are useful and advantageous are being uncovered (9). Traditional Persian medicine (TPM) with holistic approaches towards human health has utilized many herbal therapies. The French consumed herbal extracts for the eradication of PCOS symptoms (10). Natural polyphenols have been used for decades to manage hormonal problems (11). Many studies, including randomized controlled trials, case studies, and animal experiments, have been conducted on herbal medications because of their fewer side effects and to validate their traditional uses (12).

## Method

Different data bases such as Google scholars, Pubmed and Web of Science were searched for phytochemicals and plants pharmacologically validated for the prevention and treatment of PCOS. Key words used for searching included but not limited to “PCOS, “polycystic ovary syndrome”, “phytochemicals used for pcos”, “plants for PCOS”, “herbs for PCOS”, “treatment of PCOS” and “prevention of PCOS”. From the available full text articles, a master list of phytochemicals and plants was first prepared. These research studies were qualitatively evaluated for the positive and negative controls, dose, duration of therapy, method of induction of PCOS, mechanism of action and efficacy of chemical/crude drugs. The names of medicinal plants were confirmed from www.theplantlist.org.

### Pathophysiology of PCOS

In spite of the ongoing research for better understanding of the morphology, pathophysiology, and therapeutic methods of PCOS, the underlying aetiology of PCOS is still a mystery (13). A number of factors, including genetics, lifestyle, and individual characteristics, interact to cause PCOS, which in turn causes serious health consequences and a disturbed hormonal and metabolic cycle (12). The menstrual cycle is disturbed at multiple stages in women with PCOS (14). The follicular cells generate an anti-androgen called anti-Mullerian hormone (AMH), which is highly concentrated in primordial and antral follicles, and regulates their progression into the initial level. When the AMH hormone is secreted in excess, it throws off the ovarian follicle growth cycle (15). Infertility may result from immature follicles because they block the sensitivity to follicle-stimulating hormone (FSH), preventing continued follicle development (16). Overproduction of gonadotropin releasing hormone is the second most prevalent symptom of PCOS (17). This is caused by a malfunction in the feedback mechanism of the hypothalamus-hypophysis-ovarian axis (HHOA) (18). On top of that, HHOA causes an abnormally high ratio of blood luteinizing hormone (LH) to FSH (19). Because of the imbalance in the levels of these two hormones, the ovarian theca cells produce an excessive amount of the male hormone androgen (20). It leads to a reduction in the levels of oestradiol and progesterone, which induces gonadotropic hypothalamus cells to produce an increased quantity of GnRH and LH (21). The disorder’s growth and progression are thus largely attributable to this recurrent cycle of dysfunction (22).

**Figure 1:**
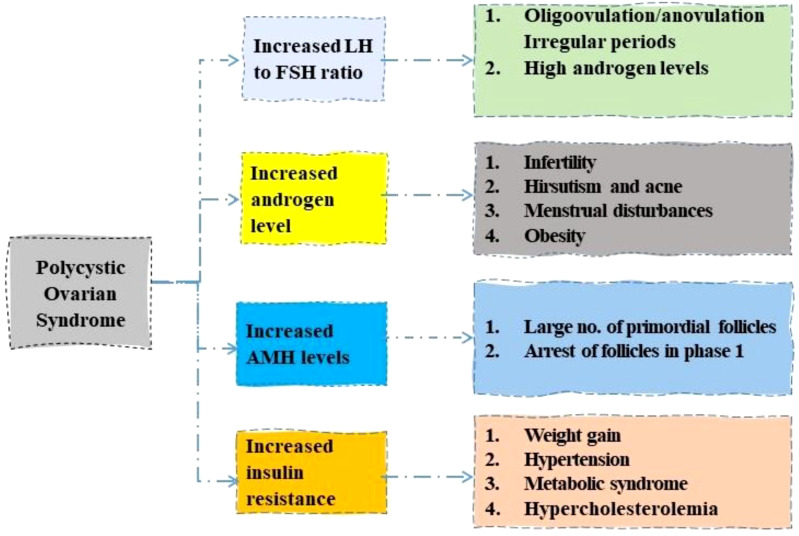
Role of different hormones in development of Polycystic ovary syndrome. LH, luteinizing hormone; FSH, follicle stimulating hormone; AMH, anti-Mullerian hormone.

The PCOS shows a marked rise in LH to FSH ratio, increased androgen and AMH levels, and insulin resistance (23). A rise in the ratio of LH to FSH levels is characterized by anovulation and irregular periods (24). Increased androgen levels in the body lead to multiple symptoms such as acne, menstrual disturbances, and obesity (25). A large number of primordial follicles are seen with an increase in AMH level (26). Obesity, high blood pressure, and metabolic syndromes are connected with PCOS with increased insulin resistance as shown in **Figure 1** (27).

### Symptoms and complications of PCOS

The PCOS exhibits its symptoms through various signs that include menstrual irregularities and a number of cysts in the ovary, causing abdominal pain and cramps during periods (28). The physical appearance also shows some abnormal changes in PCOS, such as hirsutism, acne, alopecia, and seborrhoea (29).

### Hirsutism

As a result of an increased androgen concentration, the condition known as hirsutism causes an abnormally large amount of terminal hair to grow in areas of the body that are normally bald or have only a small amount of hair growth (30). Androgenic substances such as testosterone, dihydrotestosterone (DHT), dehydroepiandrosterone sulphate (DHEAS), and androstenedione are all released by a female body when it reaches reproductive age (31). Only androgens and DHT have the ability to attach to the androgen receptors, which results in a changed morphology and physiology of the hair follicles (4).

### Acne and seborrhoea

The sebaceous glands are a type of tiny gland that secretes oil and are found in males and females both (32). The sebaceous glands are notoriously sensitive to changes in the concentration and secretion of androgens (33). In patients with polycystic ovary syndrome (PCOS), an increased concentration of androgens causes sebaceous glands to be stimulated into producing more sebum, which in turn results in the formation of acne and seborrhea (34). Androgens are to blame for the development of sebocytes, which occurs most frequently in the region of the forehead, chin, and middle back (35). The local bacteria cause an infection of the hair follicle structure and then secrete lipolytic enzymes. This causes triglycerides to break down, which in turn causes a blockage and the deposition of oil and other materials that have been destroyed in the area (4).

### Androgenic alopecia

Androgenic alopecia is a condition in which a person experiences continuous hair loss in a discernible pattern as a result of an increased concentration of androgens as well as secretions of these hormones (36). In hair follicles that are vulnerable to the process, testosterone is changed into a compound called dihydrotestosterone (DHT). This compound, then attaches to the androgen receptor as well as the genes that are responsible for the reduction in size of large terminal hair follicles over time (37). This was further supported by the results published by Cela et al., which found that 67% of the women who had alopecia, also had PCOS and an increased level of androgens in their bodies (4).

### Onycholysis and onychorrhexis

Onycholysis refers to the process in which the nail plate becomes detached from the nail bed as a result of an onychocorneal band (38). Onychorrhexis, on the other hand, refers to the process in which nails split into lengthwise bridges (39). Although the precise explanation for this result is not entirely clear, a number of studies have found that elevated androgen levels in PCOS patients are associated with more severe cases of onycholysis and onychorrhexis (2).

#### PCOS and associated disorders

PCOS patients are at higher risk for several other chronic and serious health conditions such as insulin resistance, obesity, Type 2 diabetes mellitus, coronary artery disease, atherosclerosis, hypertension, depression, anxiety and low-level inflammation. PCOS is a metabolic disorder that is greatly aggravated by obesity. Adipokines released by adipocytes cause insulin resistance which causes ovarian androgen production as well as alteration in lipid homeostasis (40). It has now been established that hyperinsulinemia adversely affects preantral follicular leading to ovulatory dysfunction (41). Metabolic changes in PCOS, such as hyperinsulinemia, hyperlipidaemia and obesity, increase the risk of cardiovascular diseases. A huge number of PCOS patients also suffer from anxiety and depression which occur due to distresses caused by symptoms such as hirsutism, obesity and altered physical appearances (42).

#### Pharmacotherapy for PCOS

**Figure 2:**
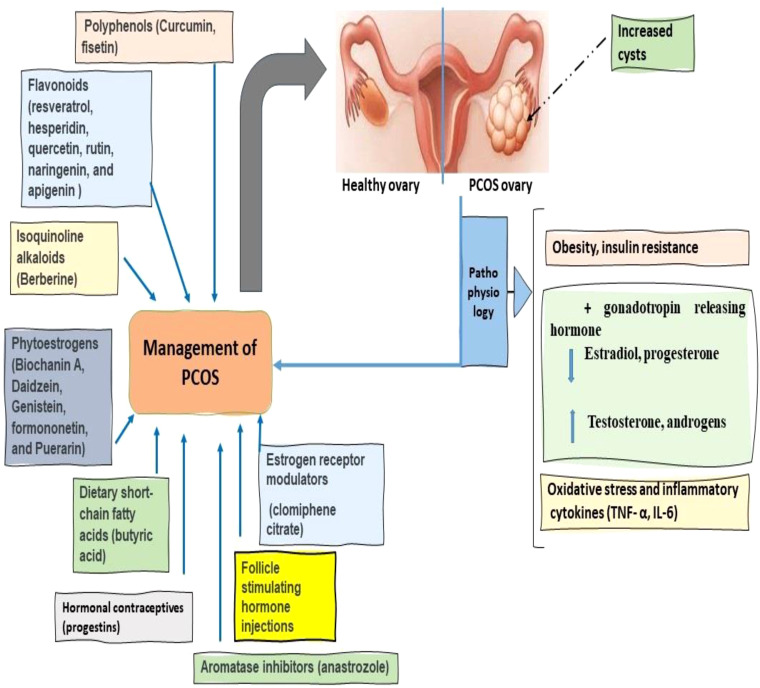
Pathophysiology and management of Polycystic ovary syndrome (PCOS).

Several drugs used for the treatment of PCOS are not devoid of harmful effects on patients (43). The issues that are linked with clomiphene citrate (CC) are also something that should be taken under consideration. These concerns include the potential danger of ovarian cancer, the antiestrogenic effects it has on the endometrium, as well as the issue of the early LH surge (44). After using CC for an extended period of time, more than 12 treatment cycles, there is a possibility of developing ovarian cancer. As a result, this medicine should only be used for a maximum of six cycles (45). Moreover, clomiphene and letrozole do not exhibit anti-estrogenic effects on the endometrium and cervical mucus (46). Dehydration, excessive urination, vomiting, headache, lethargy, gastritis, and ovarian disruption leading to irregular menstruation are some of the side effects that may be caused by using spironolactone (47). Metformin use is linked to a wide range of adverse effects, including but not limited to nausea, vomiting, digestive disturbances, and ketoacidosis (48). Furthermore, in patients with PCOS, metformin therapy must be continued indefinitely because stopping the medication after a period of three months has the potential to reverse all the disease progress (49). A long-term intervention is required for PCOS treatment owing to the significant and severe adverse reactions of currently used drugs, which warrants the search for novel therapies for the chronic management of PCOS (50). For the treatment of PCOS, the use of plant-based medications, which have fewer potential negative side effects, is likely to become the most effective strategy (2) as shown in **Figure 2**.

#### Treatment for PCOS-related symptoms

It has been shown that making changes to one’s lifestyle, such as maintaining a healthy weight loss, increasing physical activity, and making adjustments to one’s food, can help in the management of PCOS (2). Spironolactone, metformin, and eflornithine are the drugs that are considered to be the first line of defence in the fight against hirsutism (51). Because they have a low androgenic effect, additional contraceptive pills and their combination of either progestins or norgestimate, desogestrel or drospirenone are frequently used to treat hirsutism (52). Contraceptive pills, spironolactone, acarbose, and rosiglitazone are the four most common medications used to treat menstrual problems (53).

In PCOS, whole ovarian hypertrophy is seen with thickened capsule (54). There is an increased number of sub-capsular follicle cysts (55–57). The main features of PCOS are:

- Increase in ovarian size
- Capsules that are thicker than 100 µm
- An increase in the amount of subcapsular follicular cysts (58).
- A deficiency in the number of corporea lutea or albicantia
- Hyperplasia of the ovarian stroma, as well as fibrosis
- Theca cell luteinization before its time (12).

#### Phytochemicals for PCOS management

Numerous studies have demonstrated the efficacy of herbal drugs and isolated phytochemicals for the management of PCOS. Several phytochemicals, such as curcumin, rutin and fisetin, regulate the reproductive cycle via reducing testosterone level, and LH to FSH ratio (59). Some phytochemicals such as Naringenin and fisetin also increase the insulin sensitivity and improve the glycaemic control, which contributes to the betterment of PCOS symptoms (60). Some essential oils such as α-thujone are antiandrogenic in nature, so they decrease the level of androgen while improving ovulatory functions (60). Puerin, fisetin, berberine reduce the features of PCOS and associated comorbidities by decreasing oxidative stress (61). **Tables 1** and **2** show the sub-clinical and clinical studies carried out on isolated phytochemicals and plants to treat PCOS.

**Table 1:** Pre-clinical studies on plants and phytochemicals effective against polycystic ovary syndrome.

Phytochemicals	Biological source	Phytochemical used	Dose	Study Design	Study Duration	Mechanism of action and Result	References
Apigenin	*Mentha longifolia*	flavonoid	20-40 mg/kg	Ovarian tissue of PCOS induced female Wistar rats	21 days	Testosterone levels drop, and the corpus luteum and the theca layer thin out.	(62)
Biochanin A	*Trifolium pratense*	Plant derived isoflavone	0.0.05, 0.5,5, 50 mM	*In vitro* model of porcine granulosa cells	Cultured in biochanin for 48 h	Inhibition of the basal progesterone by luteinised granulosa cells	(63)
Berberine	Barks of *Berberis vulgaris* and *Berberis aristata*	Isoquinoline Alkaloid	150 mg/kg/day	DHEA induced PCOS rats	6 weeks	Reduced levels of TLR4, LYN, PI3K, NF-kB, TNF-α, IL-1, IL-6, and caspase-3.	(64)
Butyric acid	Dietary fatty acids	Short-chain fatty acid	1.1 and 11 mg/mL	human ovarian granulosa cell line, Obese PCOS mice	Varying duration	Increased estradiol and FSH, decreased testosterone, LH and insulin, apoptosis and oxidative stress	(65)
Curcumin	Rhizomes of *Curcuma longa*	Polyphenol	100 and 200 mg/kg	Letrozole induced PCOS in female Wistar rats	15 days	Marked decrease of testosterone and increase of progesterone	(66)
Diazene	*Gunnera tinctoria*	Phytoestrogen	35 mg/kg	Adult female rats	28 days	Having fewer anteric follicles and more robust primordial and primary follicles	(67)
Fisetin	*Rhus verniciflua*	polyphenol	Letrozole + fisetin (10 mg/kg)	Female Wistar rats	15days	Normalized glucose, lipids, insulin resistance testosterone, oestradiol, and progesterone	(68)
Genistein	*G. sagittalis*	Phytoestrogen	5mg/kg i.p.	Sprague Dawley rats with premature ovarian failure	Once a day for 10 days	Decreased anteric follicles and diminished serum levels of estradiol	(69)
Hesperidin	Peppermint, orange	Flavanone glycoside	10, 22.5, 50 µM/L	preantral follicles from mice	12 days	Increased expression of PCNA, FSH-R, Bcl-2, and reduced Bax genes	(70)
Naringenin	*Citrus paradesi*	flavonoid	High fructose feed diet	Adult female Wistar rats	6 weeks	Reduction of insulin resistance and oxidative stress	(62)
Puerin	*Pueraria lobata*	isoflavonoid	100/200mg/kg	Two months old female mice given cyclophosphamide and busulfan	28 days	Increased protective on premature ovarian failure via regulation of Wntß-catenin signalling pathway and oxidative stress.	(71)
Quercetin	*Camellia Sinensis*, *Hypericum perforatum*, etc	natural flavonoid	100 and 150 mg/kg/day	DHEA induced PCOS in rats	15 days	Cystic follicle count decreases in treated groups, Decreased liver glucokinase and hexokinase, increased expression of GLUT-4 and estrogen receptors	(72, 73)
Rhamnocitrin	*Nervilia fordii*	Monomethoxyflavone	1, 10 and 100 µM	DHEA- ovarian granulosa KGN cells	1-3 days	Downregulated DHEA induced expression of PPAR-γ, p-IKB and p-p65	(74)
Resveratrol	*Vitis vinifera*	plant derived polyphenol	20 mg/kg/day resveratrol and 300 mg/kg/day metformin	DHEA induced PCOS in Female Sprague Dawley rats	6 weeks	Depicting marked regulation of steroidogenesis	(75)
Resveratrol	*Vitis vinifera*	Polyphenol	20 mg/kg/day oral	100 ng/kg tributyltin induced PCOS in rats	4 weeks	Increased E2, LH and testosterone, reduced mTOR and Akt expression	(76)
Resveratrol	*Vitis vinifera*	Polyphenol	20 mg/kg/day and or Metformin 300 mg/kg	6 mg/100 g body weight DHEA	35 days	decreased LH, LH/FSH, TNF-α and tissue AMH	(75)
Rutin	*Eucalyptus macrorhyncha*	citrus flavonoid	20 mg/kg	letrozole induced PCOS in rats	2 months	Reduced level of testosterone, LH hormone and insulin resistance and ovarian tissue pathological changes	(3)
Thymoquinone	*Nigella sativa*	Essential oil	Mifepristone induced PCOS	Adult female rats	PCOS rats and Ovarian granulosa cell line KK-1	Decreased autophagy through up-regulation of androgen receptors and proinflammatory cytokines, and up-regulation of aromatase enzyme	(77)
Extract of Indian Barberry	*Berberis aristata*	Chlorobenzene, phenolic acids,	HFD+plant extract at 125 to 500 mg/kg/day	Adult female Wistar rats	45 days	Decreased insulin, leptin, hyperlipidaemia, and oxidative stress, normalized estradiol	(78)
Extracts of Flax seeds and Spearmint	*Mentha spicata L.* *Linum usitissimum L.*	Phytoestrogen	40 mg/kg HCl extract of spearmint and 200 mg/kg flax seed extract	Female Sprague Dawley rats	Given for 30 days after 7 weeks of estradiol injection	Improved endocrine profile and histomorphometry features of ovary	(79)
Aqueous seed extract of grey nicker	*Guilandina bonduc L*	Phenolic and flavonoid compounds	Letrozole 1 mg/kg+ 100, 200 and 300 mg/kg	Adult Wistar rats	30 days	Regulated menstrual cycle	(80)
Leaf extracts of sacred fig	*Ficus religiosa*	Volatile compounds	Letrozole 1 mg/kg+ leaf extract	Adult female Wistar rats	30 days	Up-regulation of CYP19a1 and PPAR-γ, reduction of oxidative stress	(81)
Whole extract and fractions of basil leaves	*Ocimum kilimandscharicum*	Phenolic and flavonoid compounds	Letrozole 1 mg/kg+ fraction or extract at 100 mg/kg/day	Adult female Wistar rats	10 days	Normalization of insulin, lipids and sex hormones, reduction of oxidative stress and increase in VEGF	(82)
Hydro-ethanolic extract of sea spinach	*Tetragonia tetragonioides (Pall.) Kuntze*	polysaccharides, sphingosine and others	Letrozole 1 mg/kg+ 100, 200 and 300 mg/kg	Adult Wistar rats	28 days	Reduced testosterone	(83)
Seed extract of bitter cumin	*Centratherum anthelminticum*	kaempferol-3-pcoumaroylglucoside, ferulic acid, and malvidin-3-(6-caffeoyl)-glucoside	HFD+ oral estradiol valerate 4 mg/Kg; extract for treatment at 250-750 mg/kg/day	Adult female rats	28 days	Decrease in oxidative stress and inflammation, normalized glucose, lipid, insulin and sex hormones,	(84)
Essential oil and α-thujone	*Thuja occidentalis L.*	Monoterpenes, fenchone	Letrozole 1 mg/kg+ T. occidentalis or α-thujone	Adult female Wistar rats	21 days	Decrease in LH, testosterone, leptin and glucose	(85)
Aqueous extract of roots	*Angelica sinensis (Oliv.)*	Phenolic and flavonoid compounds	HFD+ Letrozole+ plant extract 2, 4 and 8 g/kg	Adult female Wistar rats	30 days	Ameliorated PCOS through regulation of gut microbiota by modulating PPAR and MAPK	(86)
Aqueous ethanolic extract of seeds	*Nigella sativa*	Essential oil	1-100 µg/mL	Oocytes of mice treated with DHEA	24 h	Decreased COX-2 expression and oxidative stress	(87)

Bcl-2, B-cell lymphoma 2; DHEA, Dehydroepiandrosterone; HFD, High-fat diet; PCOS, polycystic ovary syndrome; PPAR, peroxisome proliferator-activated receptor; MAPK, mitogen-activated protein kinase; PCNA, Proliferating cell nuclear antigen.

**Table 2:** Clinical studies on plants and phytochemicals effective against Polycystic ovary syndrome.

Phytochemical	Chemical class	Source	Subject	Dose	Method	Mechanism	Result	References
Curcumin	Polyphenols	Turmeric	Women with PCOS.	500 mg thrice a day for 12 weeks	Randomized, double blind placebo control study.	Improved blood sugar and hyperandrogenism.	Decreased androgen level, Increased oestradiol level	(88, 89)
Cinnamaldehyde	Aldehydes	Cinnamic bark	15 women with PCOS	333 mg thrice a day for 8 weeks	Randomized control trial	Increased the PIK-3 activity in insulin signalling pathway	Decreased insulin resistance	(90)
Resveratrol	Polyphenol	Roots of white *Polygonum cuspidatum*	78 patients	1000 mg for 3 months	Clinical trial	lowering CYP12 and CYP21 expression and activities Inhibit Androgen production	Increased menstruation, decreased hair loss	(2, 91)
Quercetin	Flavonoid	Citrus fruits	84 PCOS women	500 mg twice a day for 12 weeks	Adiponectin mediated insulin sensitive-ty	Inhibit PIK-3	Decreased testosterone	(2)
Hesperidin	Bioflavonoid	Citrus fruits	49 subjects	500 mg BD with lifestyle changes	Randomized control trial	Nuclear factor pathway is the molecular mediators of anti-inflammatory effects.	Decreased waist circumference	(2, 92)
*Nigella sativa* oil	Essential oil	Seeds of black cumin	84 PCOS patients with oligo-amenorrhea	1 g/day for 16 weeks	Randomized, double blind study	Increased LH, testosterone, and insulin level	Increased frequency of menstrual cycle	(93)
Phytoestrogen	Isoflavone	Soy based product	Women with PCOS	Genistein 37.5mg Daidzen 10 mg Glycitein 2.5 mg for 12 weeks	Randomized, double blind study	Structurally and chemically similar to oestrogen thus binds to oestrogen receptors	Decrease free androgen index Increase free TG and plasma glutathione.	(2)
*Vitex agnus-castus*	Flavonoids	Chaste tree	Women trying to conceive	500-1000mg dried berries and 1-4ml of tincture from dry leaves for 3 months	Clinical trial	Binds to dopamine receptors and decrease cAMP and prolactin level	High level of mid-luteal progesterone and regular menstruation	(4, 94, 95)
*Cinnamomum cassia*	Terpenoids and glycosides	*Cinnamomum cassia*	15 overweight women who had oligo/amenorrhea and polycystic ovaries.	extract 333 mg 1 tablet three times a day	Double blinded, placebo controlled randomised	Reduce insulin resistance	Treatment group members showed increased insulin sensitivity. No difference was seen in body mass index, testosterone, or oestrogen levels between the two groups	(94)
Pomegranate juice	Polyphenols	Pomegranate fruit	92 women with PCOS	2L juice weekly	Randomized triple blind control trial	Reduces oxidative stress	Insulin resistance, BMI and waist circumference were reduced significantly	(96, 97)
Spearmint tea	Phenols Essential oils	Leaves of spearmint	Women with PCOS and idiopathic hirsutism	Cup of tea twice for 30 days	Clinical trials	Antiandrogenic action	Reduced free and total testosterone Cut down ovarian cysts	(98)
Catechins	Antioxidants	Green tea	34 obese Chinese women with PCOS	Green tea capsules for 3 months	Clinical trial	Binds with dopamine receptors decrease cAMP and prolactin	Weight reduction	(96, 99)
*Tribulus terrestris*	Hydroalcohol	*T. terrestris*	Women with PCOS	1000 mg of hydroalcoholic extract every day	Randomized control study	Normalizing hormone level and induce ovulation	increased FSH level, decreased glucose and LDL	(90)

cAMP, Cyclic adenosine monophosphate; CYP, Cytochrome p450 enzyme; FSH, Follicle stimulating hormone; LDL, Low-density lipoprotein;, PCOSm polycystic ovary syndrome; TG, Triglycerides.

#### Polyphenols

Polyphenols are naturally occurring chemicals that serve as a defensive mechanism against a variety of stressors (100). Because of their powerful cardioprotective, antiproliferative, antioxidative, antiapoptotic, and anti-inflammatory capabilities, the significance of such polyhydroxyphenols has significantly increased in recent years (11). Polyphenols have been shown to be effective against a wide variety of diseases, including human cancers, heart problems, cognitive problems, diabetes, metabolic disorders, aging, and inflammation-associated illnesses, as well as PCOS (101). These findings have been reported in a number of studies. Polyphenols are being investigated as a potential treatment PCOS. This is due to the fact that oxidative damage, metabolic, and endocrine abnormalities all play a significant part in the development of PCOS (11). Curcumin and fisetin are two phytoconstituents that have demonstrated a significant reduction in the symptoms of PCOS (66). The lipids and hormonal profiles as well as insulin resistance are improved by these agents. In addition, the activity of enzymes that fight free radicals is increased by these plants (102).

#### Curcumin

Turmeric contains curcumin, which is biologically active. It possesses a variety of pharmacologic actions and has the potential to be used in patients suffering from PCOS (88). Since, its isolation from *Curcuma longa* in 1815, the polyphenol curcumin has attracted the interest of researchers all over the world due to its promising biological activities (89). In PCOS patients, curcumin was useful in reducing blood glucose levels, insulin resistance and hyperandrogenism (103). In a controlled double-blind study, PCOS patients were randomly assigned to receive either curcumin (500 mg t.i.d.) or a dummy pill for a period of twelve weeks. Primary outcome indicators included fasting plasma glucose (FPG), fasting insulin (FI), sex hormone levels, and hirsutism. Secondary indicators were anthropometric measurements. Among 72 randomized people, 67 patients finished the trial (104). Once the trial is completed, fasting plasma glucose and DHEA levels had dropped much more in the treated group in comparison to the control group and the placebo (88). A statistically non-significant rise in oestradiol levels of the treatment group was evident as compared to the control group without any adverse events (88). In patients with polycystic ovary syndrome (PCOS), hyperandrogenism and hyperglycaemia may respond favourably to the use of curcumin as a dietary supplement. The findings of this research need to be supported by more extensive studies examining a wider range of dosages over longer time periods (88).

#### Flavonoids

Large amounts of flavonoids may be able to cure a variety of ailments without causing any severe adverse effects (105). Flavonoids increase the serum levels of FSH, and sharply decrease the serum levels of LH, testosterone and insulin in the PCOS-insulin resistant rats via partly inhibiting the activation of JAK2/STAT3 pathway, partially up-regulating the IL-6 expression and down-regulating the suppressor of cytokine signalling 3 (SOCS3) expression in ovaries of PCOS rats (106). Flavonoids may be natural, citrus, or bioflavonoids (107). Flavonoids extracted from various plants are found to decrease testosterone levels, improve insulin resistance and increase the expression of IL-6 to treat PCOS rats (108). Several major flavonoids are used for PCOS management, namely resveratrol, hesperidin, quercetin, rutin, naringenin, and apigenin (109). These flavonoids decrease the levels of testosterone in the body. They also improve the insulin resistance and decrease the number of cysts in the ovary (110).

### Resveratrol

Resveratrol is a plant-derived flavonoid that was initially extracted from the roots of white hellebore in 1940 and in 1960 from the roots of *Polygonum cuspidatum*, and is currently discovered in approximately seventy different kinds of herbs. Its effectiveness in the treatment of PCOS is well-established (2). It was discovered that resveratrol inhibited the production of CYP17 and CYP21 proteins as well as their enzyme activity to prevent excessive production of androgens in PCOS animals (91). Several research studies demonstrated that resveratrol exhibited PCOS ameliorating effect through decreased LH, LH/FSH, TNF-α and tissue AMH in diseased animals. In addition, resveratrol also increased E2, LH and testosterone, reduced the expression of mTOR and Akt in ovarian tissues to regulate reproductive function in female rats (75, 76). For the purpose of determining whether or not resveratrol is effective in treating PCOS, a clinical trial was carried out on 78 individuals who received 1000 mg of the medication every day for a period of three months. After therapy, the results showed an increased menstruation rate and a decreased rate of hair loss, and the levels of lipids, insulin, and androgens remained the same (2).

### Quercetin

Quercetin is an organic flavonoid that is derived from a variety of fruits and vegetables, the majority of which are citrus fruits, such as red grapes, onions, apples, berries, nuts, and seeds (111). Tea is also a good source of quercetin (112). Oxidative stress is thought to be the primary component that leads to PCOS (113). The mechanism of action is an improvement of the NF-κB signalling pathway and a correction of the inflammatory environment of the ovarian tissue (114). PCOS has also been linked to dysfunction of the endocrine glands (115). Beside affecting insulin resistance, quercetin also alters gene expression of GLUT-4 and ER in diabetic pregnant mice to improve embryo development (116). Quercetin also decreased insulin resistance in PCOS rats via decreasing liver glucokinase and hexokinase, and increasing the expression of GLUT-4 and estrogen receptors (73). In women with PCOS, their levels of adiponectin tend to be lower, regardless of their weight (117). Eighty-four PCOS women were given quercetin, and their adiponectin-mediated insulin sensitivity was tested. When compared to placebo, taking quercetin 500 mg twice per day for 12 weeks resulted in a rise in total adiponectin by roughly six percent and an increase in the level of high molecular weight adiponectin by approximately four percent. There was a drop in the level of testosterone, LH and insulin resistance in the quercetin group, which emphasized the function of quercetin in re-modelling the adiponectin-mediated insulin resistance and hormonal level in PCOS women (2).

### Hesperidin

Hesperidin is a bioflavonoid, initially extracted from the innermost layer of orange peel. It can be found in high concentrations in a wide range of citrus fruits, the most notable of which are lemon, lime, orange, and grapefruit (118). Hesperidin exhibits multiple activities, including anti-inflammatory, anti-microbial, and anti-diabetic effects (119). It appears that signalling pathways, specifically the nuclear factor pathway, are the molecular mediators of anti-inflammatory effects. However, the exact mechanism is not known (92). In a previous *in-vitro* study, it increased the maturation and growth of preantral follicles of mice to improve fertilization and embryo development via upregulating the expression of PCNA, FSH-R and Bcl-2, and downregulating Bax gene (70).

#### Isoquinoline alkaloids

Some isoquinoline alkaloids alleviate insulin resistance and activate the insulin signalling pathway. Berberine is a major member of this family (120). It promotes the utilization of glucose and reduces the level of serum androgen. It improves sex hormone binding globulin levels (121). As an isoquinoline alkaloid, berberine is the main effective component of this class, as a multi-target, multi-path plant constituent that interferes with the development of PCOS and related comorbidities with a few adverse reactions (122). It was found that berberine reduced the expression of toll-like receptor 4 (TLR4), Src family tyrosine kinase (LYN), phatidylinositol 3-kinase (PI3K), NF-κB, TNF-*α*, IL-1, and caspase-3, which was accompanied by a reduction in cell apoptosis, which pointed to the potential significance of berberine in the therapy of PCOS (123).

#### Phytoestrogens

Phytoestrogens are substances that naturally occur in plants. They have a similar chemical structure to our own body’s oestrogen and are able to bind to the same receptors as that of oestrogen (124). Phytoestrogens have shown both estrogenic and anti-estrogenic effects (125). Isoflavones are the most widely studied phytoestrogens. These are abundantly found in soybeans, legumes, berries, grains, nuts, and wine. Resveratrol, found in fruits, berries, red wine, chocolate, and peanuts, is believed to be responsible for some of the health benefits that include improved hormonal profile, insulin resistance, and antioxidant effects (126).

Phytoestrogens are isoflavones commonly found in soya products. There is evidence that phytoestrogens like Biochanin A, Daidzein, Genistein, formononetin, and Puerarin can help in the management of PCOS symptoms (2). PCOS women experience a disruption in the process of the production of androgen as well as its metabolic process and oestrogen, which causes an increase in the concentrations of androstenedione, testosterone, and dehydroepiandrosterone in their serum (2). Phytoestrogens have molecular structures and sizes nearly identical to oestrogens such as 17-estradiol and diethylstilboestrol. As a result, these attach to oestrogen receptors and have anti-estrogenic effects, helping PCOS patients with hormonal imbalances (2). A randomized, double-blind, and placebo-controlled trial was carried out to demonstrate the effectiveness of soy-isoflavone and phytoestrogen against PCOS. Phytoestrogens consisting of 37.5 mg genistein, 10 mg daidzein, and 2.5 mg glycitein were given to PCOS patients for a period of 12 weeks. A considerable drop was seen in both the free androgen index, serum triglyceride, and insulin levels as a result of therapy with phytoestrogens. Additionally, a significant rise in plasma glutathione level and a fall in monoaldehyde level pointed towards a favourable effect of phytoestrogens on the management of PCOS (2).

#### Catechins

Green tea is derived from the plant *Camellia sinensis* and contains a high concentration of catechins along with several minerals and vitamins (127). It has several health benefits, including but not limited to effectiveness against diabetes, insulin resistance, and obesity (96). A total of thirty-four obese Chinese PCOS patients were selected at random to receive either treatment with green tea capsules or a placebo for a period of three months. After receiving therapy, each group’s anthropometric measurements and biochemical and hormonal profiles were compared to those taken before treatment. After treatment, little but noticeable weight loss among those who had consumed green tea without any noticeable variation in hormone levels (99). Various preclinical studies on individual phytochemicals and plants effective against PCOS are shown in **Table 2**.

### Polyunsaturated and dietary short-chain fatty acid

Previous studies have shown that a decline in carnitine, a metabolic intermediate of fatty acids, causes oocyte maturation and regulates energy metabolism and transport of fatty acids (128). Polyunsaturated fatty acids, such as omega-3 and α-linolenic acid play important therapeutic role in PCOS through reduction of inflammation and oxidative stress, and normalizing hormonal irregularities (129). In addition, short-chain fatty acids including butyric acid, are usual breakdown products of dietary fibres by gut microbes, which affect cellular functions such as apoptosis, proliferation and adiposity by G-protein-coupled and other receptors. Butyric acid inhibited PCOS symptoms through reducing insulin resistance, ovarian inflammatory cytokines and gut microbiota (65). Butyric acid treatment of human granulosa tumour cells ameliorated lipopolysaccharide-induced apoptosis, inflammatory cascade and oxidative damage. In addition, intraperitoneal administration of butyric acid exhibited a decline in follicular count, LH, testosterone and insulin level, and increased FSH and estradiol level in obese PCOS mice (65).

#### Plants to treat PCOS

Several medicinal and dietary herbs and plants have been investigated for the management of PCOS (130). Several plants such as *Vitex agnus*, *Cinnamomum* genus, pomegranate, *Tribulus terrestris, Mentha* species and *Nigella sativa* have been investigated for their sub-clinical and clinical effectiveness to manage and treat PCOS symptoms.

### Vitex agnus-castus

The *Vitex agnus-castus* (VAC) plant is a shrub or a small tree. The flavonoids make up the majority of VAC components. *In vitro* research has demonstrated that the flavonoids such as castidin, quercetagetin, and isovitexin have an effect on oestrogen receptors (95). VAC has been shown to be efficacious in both pre-clinical and clinical study for the reduction of prolactin levels, the improvement of the menstrual cycle, and the treatment of infertility. Compounds in VAC binds to dopamine type 2 (DA-2) receptors in the CNS, decreasing cAMP and prolactin release (94). In previous research, women who had been trying to conceive for six to thirty-six months were given a supplement containing VAC (4). It is recommended to take between 500 and 1000 mg of dried berries and 1–4 ml of a tincture made from dried plants. After three months, the supplementation group showed significantly higher levels of mid-luteal progesterone and regular menstrual periods, in contrast to the placebo group, which showed no significant changes. 14 of the 53 women who took the supplement became pregnant, compared to only 4 of the placebo group’s 40 women who did not become pregnant (4).

### Cinnamomum genus

As a condiment and aromatic plant, cinnamon is widely used. Its bark and leaves are used to make cinnamon oil. Its major bioactive components include polyphenols and cinnamaldehyde (131). It has been proven that the PCOS-treating properties of *Cinnamomum zeylanicum* include both reproductive and metabolic benefits (90). It was observed that cinnamonaldehyde had decreased the blood glucose through upregulating the expression of the GLUT4 gene. Cinnamaldehyde also increased the antioxidant response of reactive oxygen species produced in hyperglycaemia so as to safeguard pancreatic beta cells (132). PCOS patients receiving an extract of cinnamon three times a day for eight weeks experienced an improvement in their insulin sensitivity (133). The process for reducing insulin resistance involves increasing glucose utilization and potentiating the insulin signalling pathway via phosphatidylinositol 3-kinase (PI-3 kinase) at the post-receptor level. Recent clinical trial findings show that menstrual disruption in women with PCOS may benefit from cinnamon supplementation of 1500 mg/kg for 6 months. It is thought to be due to insulin resistance that improves and reduces menstrual irregularities (90).

The *Cinnamomum cassia* (Chinese cinnamon) tree is a tropical evergreen tree that is fragrant. Terpenoids, phenylpropanoids, and glycosides are the primary chemical components found in *Cinnamomum cassia* (134). A placebo-controlled and randomized experiment was carried out for a total of eight weeks. This study was carried out on 15 obese women who were also suffering from oligomenorrhea or amenorrhea and PCOS. Participants received either 333 mg of *Cinnamomum cassia* extract or placebo in a tablet three times per day for the entire duration of the study. Insulin sensitivity was significantly improved in the therapy group. There was no significant difference between the two groups in terms of BMI, testosterone levels, or oestradiol levels (94).

### Pomegranate juice

The majority of the phytoconstituents found in pomegranate fruit are polyphenols (96). 92 women with PCOS participated in a parallel, randomized, and triple-blinded study. Three treatment groups, each consisting of twenty-three patients, were given two litres per week of either symbiotic pomegranate juice (SPJ), pomegranate juice, or symbiotic beverage (SB). The patients in the control group were given two litres of a placebo beverage per week. At the conclusion of the research project, 86 patients were examined. Insulin resistance, BMI, weight, and waist circumference were decreased significantly in the treatment groups. Both the SPJ and SB groups demonstrated a considerable reduction in their testosterone levels. Any noticeable difference in the FPG, LH, or FSH levels between any of the groups was not evident (97).

### Tribulus terrestris

In women with PCOS, the floral parts and fruits of *Tribulus terrestris* have been shown to increase the frequency of ovulation and decrease the size of ovarian cysts (135). The treatment with 10 mg of a hydroalcoholic extract of *T. terrestris* normalized the menstrual irregularity as well as hormonal changes. Additionally, the ovarian cysts were effectively eliminated and the normal function of the ovary was restored (136). In PCOS, the postulated mechanism of *T. terrestris* was the normalization of the hormonal balance and the induction of ovulation through antiestrogenic action. In a randomized control study, women who took 1000 mg of hydroalcoholic *T. terrestris* extract every day showed promising hypoglycaemic effects. Treated patients also had less total cholesterol and low-density lipoproteins, which showed therapeutic effectiveness of this crude drug against obesity-induced PCOS (90).

### Mentha species

*Mentha spicata* (commonly known as Spearmint) and *Mentha piperita* (known as peppermint) are among the most widely studied species of Lamiaceae family for the treatment of PCOS (137). Both species have been evaluated for different reproductive health problems in women such as PCOS, amenorrhea and dysmenorrhea. These species have two major bioactive components; essential oils and phenols (138). Studies on spearmint extract demonstrated that it improved PCOS symptoms and ovarian histology against estradiol and letrozole induced PCOS disease models (139). Spearmint oil also increased follicular development at 150 and 300 mg/kg dose in PCOS rats through decreasing ovarian cysts, atretic follicles and testosterone level (140). *Mentha piperita* herbal tea (40 g/L) demonstrated the efficacy to treat PCOS in letrozole induced PCOS in rats and moderated letrozole induced fibrosis in ovary by decreasing estradiol level (139). Clinical trials showed that drinking spearmint tea (5 g in 250 mL water) twice a day for 30 days reduced testosterone levels (both free and total), and raised levels of LH and FSH. It is possible to draw the conclusion that *Mentha spicata* is a useful antiandrogenic treatment in patients diagnosed with PCOS because it lowers both free and total androgen levels and cuts down on the number of ovarian cysts (98).

### Nigella sativa

Nigella sativa, also known as black cumin or black seeds, has been widely documented for its potential to treat PCOS. A previous study demonstrated that seeds extract of nigella improved the maturation of oocytes isolated from PCOS mice by inhibiting the expression of COX-2 and oxidative stress (87). The efficacy of Nigella sativa oil was later confirmed in PCOS patients exhibiting oligo-amenorrhea. Nigella oil increased menstrual regularity in PCOS patients by increasing LH, testosterone and insulin level in PCOS patients (93). Thymoquinone, an essential oil found in *Nigella*, showed its effectiveness against mifepristone induced PCOS in rats by decreasing autophagy through up-regulation of androgen receptors and proinflammatory cytokines, and up-regulation of aromatase enzyme (77).

### Adverse effects and toxicity of phytochemicals

Use of phytochemicals and plant extracts is not devoid of adverse effects. It is found that long-term use of phytoestrogens such as genistein, disrupts the pituitary-hypothalamus axis resulting in long-term inhibition of endogenous steroid production (141). It is well-established that oestrogens increase the risk of breast cancer, early onset of maturity, and gravidity in exposed individuals. However, the association of these adverse effects with phytochemicals is still to address. Some phytochemicals, such as genistein can worsen hypertension due to their vasopressin action (142). Abdominal pain, diarrhoea and headache are common adverse effects of phytochemical therapy. Phytochemicals and plant extracts used against PCOS need further investigation related to their safety in human (143).

## Conclusion and future prospective

Recently, the incidence of PCOS is increasing due to genetic, environmental, and intrinsic individual factors, the management of which poses a significant hurdle to better quality of life and reproductive health in female individuals. Currently available medications focus on regulating the menstrual cycle, obesity, and insulin resistance. Several isolated phytochemicals such as curcumin, berberine, rutin, resveratrol, quercetin, hesperidin, biochanin, apigenin, fisetin, diazene, genistein, formononetin, and puerin have shown potential to treat PCOS in preclinical studies. Moreover, curcumin, cinnamaldehyde, resveratrol, quercetin, hesperidin, phytoestrogen, *Vitex agnus-castus*, *Cinnamomum cassia*, pomegranate juice, spearmint tea, catechins, *Nigella sativa* and *Tribulus terrestris* have demonstrated their efficacy to treat symptoms in PCOS patients.

These isolated phytochemicals and herbal drug formulations may serve as alternatives to metformin and clomiphene citrate for PCOS management as well as be used as adjuvants to conventional therapy for synergistic effect. Therefore, further large-scale clinical studies must be carried out to evaluate the dosage and duration of therapy with these phytochemicals and herbal drugs individually and in combination with other drugs to treat PCOS. The main problem with using these phytochemicals is the limited knowledge regarding their safety profile, quality, and efficacy. Hence, there is an extensive need to emphasize research regarding the efficacy, safety, and metabolomic profiling of these herbal drugs to achieve therapeutic outcomes and assure the quality of products.

## Author contributions

SM: Data curation, Writing – original draft, Writing – review & editing. SS: Investigation, Writing – original draft, Writing – review & editing. AS: Investigation, Writing – original draft, Writing – review & editing. MK: Conceptualization, Writing – original draft, Writing – review & editing. AK: Formal analysis, Writing – original draft, Writing – review & editing. MA: Conceptualization, Supervision, Writing – original draft, Writing – review & editing.
